# The Complex Interplay of Genetic and Lifestyle Risk Factors in Type 2 Diabetes: An Overview

**DOI:** 10.6064/2012/482186

**Published:** 2012-10-08

**Authors:** Paul W. Franks

**Affiliations:** ^1^Genetic & Molecular Epidemiology Unit, Department of Clinical Sciences, Skåne University Hospital, Lund University, 205 02 Malmö, Sweden; ^2^Department of Nutrition, Harvard School of Public Health, Boston, MA 02115, USA; ^3^Genetic Epidemiology & Clinical Research Group, Section for Medicine, Department of Public Health & Clinical Medicine, Umeå University, 90186 Umeå, Sweden

## Abstract

Type 2 diabetes (T2D) is one of the scourges of modern times, with many millions of people affected by the disease. Diabetes occurs most frequently in those who are overweight or obese. However, not all overweight and obese persons develop diabetes, and there are those who develop the disease who are lean and physically active. Certain ethnicities, especially indigenous populations, are at considerably higher risk of obesity and diabetes than those of white European ancestry. The patterns and distributions of diabetes have led some to speculate that the disease is caused by interactions between genetic and obesogenic lifestyle factors. Whilst to many this is a plausible explanation, remarkably little reliable evidence exists to support it. In this review, an overview of published literature relating to genetic and lifestyle risk factors for T2D is provided. The review also describes the concepts and rationale that have motivated the view that gene-lifestyle interactions cause diabetes and overviews the empirical evidence published to date to support this hypothesis.

## 1. Introduction

T2D imposes an extraordinary burden on affected individuals, their families, and on society as a whole. Diabetic complications are often severe, reducing quality of life and productivity, and causing premature death in many patients. By the year 2030, more than 400 million people globally are projected to have developed T2D [1]. The rising prevalence of diabetes is thought to be driven by four key factors: (i) ageing societies, (ii) improved survival of diabetic patients, (iii) improved surveillance, and (iv) increased prevalence of obesogenic behaviors. Of these explanations, the last, being the only modifiable factor, is unsurprisingly an area of intense discussion and calls to action amongst diabetes policy makers and practitioners.

T2D is diagnosed on the basis of repeated elevated venous blood glucose concentrations when fasted (≥8.0 mmoL/L) or 2 hrs following an oral glucose challenge (≥11.1 mmoL/L), or elevated (≥6.5%) glycosylated hemoglobin (HbA1c) levels [2], which are unexplained by other known causes. Insulin is the primary hormone responsible for relocating glucose from the blood into the body's cells. In a healthy resting person, insulin production generally increases soon after glucose is ingested, which initiates a progressive decline in blood glucose concentrations toward or below the fasting levels. However, when insulin production is diminished, blood glucose remains elevated for much longer. Although glucose is an essential substrate for the body's cells, not least because the brain is entirely dependent on glucose as a source of fuel, long-term exposure to elevated blood glucose concentrations is associated with severe damage to the heart, kidneys, and eyes, as well as the nerves in the hands and feet, although it is unclear whether this is a direct effect. 

A person's susceptibility to diabetes depends on a combination of intrinsic factors that influence (i) the insulin producing capacity of the pancreatic beta cells, (ii) cellular insulin sensitivity, (iii) the amount of glucose coming from the gut (digestion of food) and liver (gluconeogenesis), and (iv) the extent to which glycogen is degraded (glycogenolysis). Thus, T2D is caused by a relative deficiency in insulin action, with the primary cause of this deficiency relating to defect(s) in exogenous insulin production, insulin signaling, and/or the overavailability of glucose. In part because T2D is well established to have strong genetic [3] and lifestyle [4] determinants, many have speculated that the disease is caused by gene-environment interactions [5], with the “environmental” component relating primarily to lifestyle factors such as physical inactivity, poor diet, and obesity. 

## 2. Brief Overview of the Genetics of T2D

Evidence that T2D has a strong genetic component came initially from family-based studies, where the observation that diabetes clusters within groups of biologically related individuals led to the quantification of diabetes heritability [3] and familial risk, through which having parents with diabetes was determined to double the risk of T2D [6]. The Botnia Family Study provided seminal evidence of the metabolic consequences of diabetes family history; this study showed that offspring with first-degree diabetic relatives tend to be more obese and insulin resistant and have lower basal metabolic rate [7] and aerobic fitness [8] and are more prone to develop T2D [8, 9] than people without a family history of diabetes. These metabolic disturbances are thought to result from defects in skeletal muscle oxidative energy metabolism. For example, insulin resistant, but otherwise healthy, young adults with a positive family history of diabetes [10] are more prone to accumulate intramyocellular lipids, the inorganic phosphate : phosphocreatine ratio in skeletal muscle is lower, and mitochondrial phosphorylation is substantially blunted, compared with persons who are insulin sensitive, regardless of family history. 

Quantitative genetics, which exploits information about familial relatedness and disease (or trait) coalescence, has been used to estimate the extent to which a disease or trait is explained by genetic factors. “Broad sense” heritability estimates (H2) for T2D reflect the ratio of the total genetic to the total environmental variances explained by these factors for a given phenotype. In conventional quantitative genetic analyses, the genetic and environmental influences on the phenotype are estimated by comparing related and unrelated individuals, while the phenotype is directly measured. There are various factors that may mean that H2 overestimates the underlying genetic component of a phenotype; these factors include correlations between genotypes and environmental factors and the sharing of environmental factors amongst related individuals. To overcome some of these limitations, methods have been developed that allow the partitioning of genetic (*a* ^2^) from shared (*c* ^2^) and unshared (*e* ^2^) phenotypic variances. The gold-standard design includes studying identical twins separated at birth and reared apart (often called “the adopted twin pair design”). Whilst the latter is more robust to confounding than H2 estimates, genetic estimates derived using the adopted twin pair design may still be confounded by nongenetic intrauterine environmental factors, which are shared by identical twins but not by unrelated individuals. For diseases such as T2D and obesity, where intrauterine programing events impact the diseases' etiologies, this source of confounding may lead to an overestimation of the genetic influence on a particular trait. Nevertheless, heritability estimates do help researchers determine whether costly molecular genetic studies of a given trait are justified. In the case of T2D, heritability estimates derived from the Danish Twin Registry have been widely cited [3]. In this study, additive genetic (*a* ^2^) factors explained 26% and shared environmental (*c* ^2^) factors explained 41% of the twin resemblance in diabetes, with unshared environmental factors and model error (*e* ^2^) explaining the remainder of the variance. 

The search for the specific genetic variants that cause T2D has been underway since the mid-1990s. Linkage studies in families and genetic association studies in cohorts of unrelated individuals have been the predominant approaches used by population geneticists. Although linkage studies have proven highly effective for the detection of rare, highly penetrant genetic loci responsible for diseases such Huntington's and Tay-Sachs, the inherent sample size limitations of family-based studies have rendered linkage scans too small (and thus underpowered) to detect loci for T2D, which are generally more frequent, but convey relatively small effects; even meta-analyses of T2D linkage scans, for example, include <10,000 individuals [11], a sample size that is dwarfed by modern genetic association study meta-analyses that approach 100,000 case-control samples [12]. However, these very large sample collections have only recently become available, and genetic association studies conducted prior to 2007 (the point at which genome-wide studies started to gain popularity) were often confined to no more than a few hundred case-control pairs. A very noteworthy exception to the failure of linkage scans in T2D genetics is that of *TCF7L2*, which was initially identified by fine-mapping a linkage peak at Chromosome 10q25.3 [13]. Importantly, however, the T2D single-nucleotide polymorphism (SNP) that was discovered by fine-mapping the region beneath the linkage peak did not explain the linkage signal, indicating that the discovery of this T2D locus was largely serendipitous. At the time when *TCF7L2* was discovered, only two other *bona fide* T2D loci were known (*PPARG* Pro12Ala and *KCNJ11* E23K). However, the advent of massively parallel, high throughput genotyping technologies (in the form of genome-wide association studies [GWAS] and other variations on these arrays including Metabochip and the Exome chip), combined with the availability of very large case-control cohorts, and a willingness of geneticists to work closely together, triggered a quantum leap in the discovery of T2D loci. Indeed, since the discovery of *TCF7L2* in 2006 [13], more than 100 independent genetic loci have been discovered and are now reliably associated with T2D [12] or its quantitative metabolic traits (glucose and insulin) [14]. 

## 3. Brief Overview of Lifestyle and T2D Risk 

Family history of a disease implies the presence of genetic risk factors that are shared amongst family members. However, the relationship between established genetic polymorphisms and parental history of diabetes is surprisingly weak [15]. One explanation for this apparent anomaly is that diabetes-predisposing behaviors, as well as genes, are shared amongst family members. Whilst heritability studies of T2D are often used to underscore the importance genetic factors play in the etiology of the disease, they emphasize to an even greater degree the substantial role environmental risk factors play. Of the many probable environmental determinants of diabetes, obesity stands out as the most strongly established modifiable risk factor. At the point of diagnosis, roughly 80–95% of European whites with diabetes are overweight or obese [16]. Cohort studies indicate that a standard deviation unit increase in body mass index (BMI: 3.6 kg/m^2^ in men, 4.5 kg/m^2^ in women) or waist circumference (10.1 cm in men, 11.3 cm in women) equates to approximately a doubling of the hazards of developing T2D in European populations [17]. Perhaps most convincingly, clinical trials, in which participants were randomized to either standard of care or intensive lifestyle interventions, demonstrate that the risk of T2D declines in a manner that is dependent on the degree of weight loss and in the absence of weight loss, there is minimal risk reduction from diet and exercise [18]. It stands to reason, therefore, that risk factors which are causally associated with obesity are also likely to convey risk for T2D, albeit to a lesser extent (because obesity is not an absolute determinant of T2D).

Physical inactivity and sedentary behaviors are important risk factors for a number of complex, noncommunicable diseases such as T2D. Prior to the introduction of insulin, vigorous exercise was frequently prescribed to control blood glucose concentrations in people with diabetes. Studies conducted in the first half of the Twentieth Century sought to elucidate the mechanisms through which exercise lowers blood glucose concentrations in people with diabetes [19]. Studies conducted in the 1960s began to employ techniques that enabled regional and systemic peripheral glucose utilization to be compared during rest and exercise and noted “the necessity for regulation of physical activity in any therapeutic program that is designed to monitor the utilization of extracellular glucose.” [20]. However, it was not until the latter half of the Twentieth Century that the specific insulin-independent mechanism of exercise for glucose translocation from the blood into the cell nucleus was identified, with GLUT4 as the key molecule [21]. 

There is an extensive literature on dietary and nutritional risk factors for T2D. Most of the published evidence in this regard has come from epidemiologic studies. The Nurses' Health Study and the Health Professional's Follow-up Study have been the source of much of the literature on the nutritional epidemiology of T2D in women and men. Studies in these cohorts have shown that high intakes of sugar sweetened beverages [22–25], fruit juice [22], red [26, 27] and processed [28, 29] meats, white rice [30], fish [31], heme iron [32, 33], potatoes [34], transfatty acids [35], low carbohydrate [36] and high glycemic load [37–40] diets, and irregular eating patterns [41], and overall poor quality diets [42–44] are associated with increased diabetes risk. By contrast, dietary anthocyanins(a flavonoid) [45], whole grains, dietary fiber and brown rice [30, 38, 46–48], zinc [49], vitamin D and calcium [48, 50] magnesium [48, 51], potassium [48], caffeinated [52, 53] and decaffeinated coffee[52], dairy produce [54], fruit and vegetable [55], light-moderate alcohol [56–58], nuts and peanut butter [59], polyunsaturated fatty acids [35] and vegetable fat [48] intake, and healthy diet patterns combined with other healthful lifestyle behaviors [60] are associated with lower diabetes risk. Not all of these findings are adequately replicated to justify inclusion in the ADA diet recommendations for the prevention of T2D [4]. For example, a recent large European nested case-cohort study of diet and incident T2D called InterAct [61] found that although fruit and vegetable intake per se was not associated with diabetes risk, root vegetables were [62]. The same study showed no association between overall dairy intake and diabetes incidence, but found evidence to support an association of cheese and combined fermented dairy product intake with lower diabetes incidence [63]. Although the Nurses' Health Study and Health Professional's Follow-up Study [31] reported an increased risk of diabetes with higher total fish intake, the InterAct study showed that fatty fish intake was associated with lower diabetes incidence rates (63]. Whereas InterAct [64] and the Health Professional's Follow-up Study [42] both suggest that Mediterranean-style diets are associated with lower diabetes risk. 

The Cohorts for Heart and Aging Research in Genomic Epidemiology (CHARGE) Consortiumhas also yielded valuable epidemiologic data on the relationships between dietary factors and glucose and insulin concentrations. For example, cross-sectional studies from CHARGE including almost 50,000 white participants from the US and Europe showed strong inverse associations between dietary whole grains [65], total zinc intake [66], and diet pattern [67] with blood glucose and/or insulin concentrations. 

In a recent comprehensive review of the nutritional epidemiology literature, Salas-Salvado et al. [68] concluded that intake of fresh vegetables and other plant-based foods, whole grains, pulses and nuts, and reduced intake of red and processed meat, sugar-sweetened foods and beverages, high-fat dairy products, and refined grains all appear protective of T2D.

The most recent American Diabetes Association diet guidelines for the prevention and management of T2D focus primarily on diets that lead to maintainable weight loss [4], as clinical trials show that weight loss is the most impactful physiological adaptation that can be safely induced for the prevention of diabetes and other chronic diseases [5, 69]. However, the ADA also acknowledges that even though a range of diets have been shown to reduce body weight in the short term (up to 6 months), the bigger challenge is maintaining long-term weight loss, as short-term successful weight loss is invariably followed by a period of weigh regain. In a randomized controlled trial comparing four different weight loss diets that varied in their composition of fats, carbohydrates, and protein [70], the authors concluded that irrespective of the diets' macronutrient contents, a similar degree of weight loss and weight-regain occurred, and that by the end of the trial (2 yrs), the majority of participants had returned to their baseline weights. Studies such as this illustrate the difficulties in maintaining long-term weight loss even in those at high risk of diabetes, despite widespread awareness that weight gain is a major risk factor for the disease. Nevertheless, there are those who are successful in maintaining reduced weight over the long-term; it maybe that molecular biomarkers, such as genotypes, exist can be used to predict a person's susceptibility to weight regain after intentional weight loss; this might help identify those who are likely to require most support in weight loss intervention studies and are hence most prone to develop diabetes. 

## 4. Genetics of Weight Loss and Weight Regain

Weight loss and weight gain have a major role in the development of T2D. Thus, many have sought to understand the mechanisms through which these processes occur in anticipation that this might aid in the prevention of diabetes. In a recent study from the Diabetes Prevention Program (DPP) [71], a multiethnic randomized clinical trial of weight loss for diabetes prevention [69], we identified a number of genetic markers that predict weight regain after intentional weight loss through lifestyle modification or metformin treatment. Two of these variants (*BDNF* rs6265, *PPARG* Pro12Ala/rs1801282) conveyed effects on weight regain that were statistically significant after adjustment for multiple hypothesis testing and were evident in the entire multiethic cohort and in Non-Hispanic Whites only, mitigating the possibility that confounding by population stratification underlies these effects. Two other variants (*TMEM18 *rs6548238, *KTCD15* rs29941) interacted with treatment modality to affect weight regain. Novel associations with short- and/or long-term weight loss or change in adiposity were also identified for the *PPARG* rs1801282, *NEGR1* rs281575, *FTO* rs9939609, *SH2B1* rs7498665, and *MSRA* rs7826222 variants.

Although genetic associations with weight loss are biologically informative, the genetic associations with weight regain are probably most clinically relevant, as this information might help target additional support towards persons who struggle most in efforts to maintain reduced weight, thus improving the long-term efficacy of weight-loss interventions. Irrespective of which weight-loss intervention DPP participants were randomized to, variants in *NEGR1* and *BDNF* predicted weight regain in DPP participants. Both genes are expressed within the central nervous system (CNS). The mechanisms that link *NEGR1* with obesity are poorly understood. *NEGR1* is a cell adhesion molecule that is involved in establishing and remodeling the neural circuit [72] and determining synapse number in hippocampal neurons [73, 74]. Thus, it is possible that functional variants in *NEGR1* may have wide-reaching effects on the development of conditioned traits such as taste, hunger, satiation, and other preference driven aspects of energy balance behaviors [75]. *BDNF* is expressed in a range of human tissues including brain. *BDNF* phosphorylates AMP-activated protein kinase (AMPK), a key sensor of cellular energy levels, and acetyl coenzyme A carboxylase-beta (ACCbeta), processes that enhance fatty acid oxidation [76]. By consequence, *BDNF* transcription and translation increases in myocytes following electrical stimulation, suggesting that *BDNF* is exercise responsive; it has long been recognized that BDNF protects against neuronal damage inflicted by hypoxic ischemia [77], another process impacting cellular energy metabolism. BDNF transcription and germline mutations have been associated with rare syndromic forms of obesity [78, 79], and animal studies indicate may be attributable to hyperphagia caused by disruptions to the appetite and satiety regulating centers of the brain, such as the ventromedial and dorsomedial hypothalamic axes [80–82].

Several loci (*FTO* rs9939609, *TMEM18* rs6548238, *BDNF *rs6265, *KTCD15* rs29941) showed nominally statistically significant treatment-specific effects on rate of weight regain in the entire DPP cohort and in Non-Hispanic Whites only. *FTO* has been widely studied for its role in energy intake and other aspects of energy metabolism [83–93], but little is known of the mechanisms underlying the relationship of this locus with obesity in humans. The *FTO *rs9969309 variant is unlikely to be functional owing to its location within an intron, although it may participate in the transcriptional regulation of *FTO* and neighboring genes. Mice lacking the *Fto* gene are leaner than wild-type mice owing to increased total energy expenditure and sympathetic activity; spontaneous locomotor activity does not differ in these animals, however, and they are hyperphagic [94]. Elsewhere, the ratio of lean to fat mass in mice harboring a dominant *Fto* mutation increases with high fat feeding; fat and carbohydrate metabolism genes in white adipose tissue are also overexpressed in these animals [95]. As with the weight loss analyses, we were unable to detect any statistically significant effect mediators of the SNP effects on weight regain.

Almost all of the loci studied here have been associated with an obesity-related trait in recent GWAS meta-analyses [96]. The exception is the *PPARG* Pro12Ala/rs1801282 variant, which has not featured as a top-ranking variant in GWAS, but may be associated with obesity through interactions with lifestyle factors such as dietary fat intake [97]. We have previously examined this variant in relation to obesity-related traits in the DPP and identified gene-treatment interactions [98, 99]. It is possible that such interactions mask the main effect of a variant when studied in GWAS [100]. Our findings suggest that the *PPARG* Pro12Ala/rs1801282 SNP may be of significant clinical interest, as it was associated with larger baseline waist circumference, larger visceral adipose tissue (VAT) area, and with all three longitudinal obesity metrics (greater short-term and long-term weight loss and greater tendency for weight regain) in the full DPP study sample, regardless of treatment group. Nicklas et al. also found that the Pro12 Ala variant was associated with greater weight loss after a 6-month weight-loss intervention and greater regain at 1 year following the absence of continued intervention, noting reductions in the rate of fat oxidation and fat oxidation relative to resting metabolic rate [101]. Interestingly, we found that reductions in adiposity with weight loss were primarily via reductions in subcutaneous (SAT) and not VAT, indicating that genetic variation may influence which fat stores are metabolized and reduced with lifestyle or metformin intervention. We found that some loci (*NEGRI* rs2815752) specifically influenced long-term weight loss outcomes when individuals were assigned to metformin treatment. This information may be helpful when seeking to tailor choices of lifestyle or medication treatments for obesity. 

Interestingly, the *TMEM18* rs6548238 nonobesogenic allele was protective of weight regain in the lifestyle intervention, but increased the rate of regain in the placebo group, indicating that aspects of the lifestyle interventions, such as caloric restriction and alterations in the pattern of fat intake, may modify the obesogenic effect of the gene, possibly in a ligand-dependent manner. 

## 5. What is a Gene-Environment Interaction?

Before proceeding to discuss the evidence base and public health implications of gene-lifestyle interactions, it is important to begin by defining what the concept means in the framework of this review and in other settings. Here, the word “interaction” will be used in a formal statistical sense, where the combined effects of the genetic and environmental (in this case the environmental factors are lifestyle behaviors) exposures are more than additive. 

**Figure 1:**
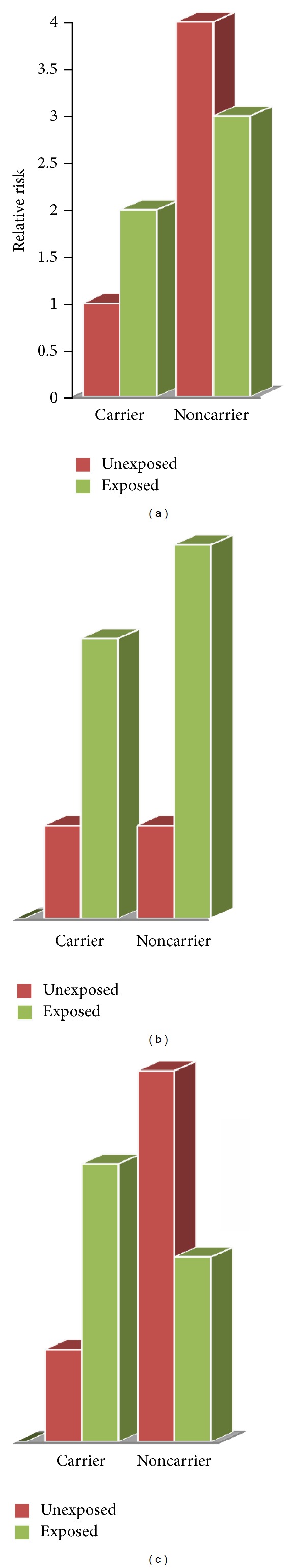
Three different types of interaction effects for a dichotomous outcome, such as diabetes. (a) shows a “removable interaction.” In this scenario, the genotype exerts an effect in people who are exposed as well as in those who are not exposed to the environmental risk factor, but larger genetic effect in one environmental exposure group than in the other. (b) shows a “nonremovable, pure interaction,” which is characterized by the presence of a genetic effect only in one of the two environmental exposure groups. (c) shows a “cross-over” interaction, where a genotype conveys risk of disease in people who are exposed to the environmental risk factor, but is protective of disease in persons who are unexposed to the same environmental factor.

In very simple terms, interaction effects can be described in one of three ways.  Figure 1 shows examples of different types of interaction effects for a dichotomous outcome, such as diabetes. The first type is sometimes called a “removable interaction” (Figure 1(a)). In this scenario, the genotype exerts an effect in people who are exposed as well as in those who are not exposed to the environmental risk factor; however, the genetic effect is substantially larger in one environmental exposure group than in the other. Importantly, the scale on which the interaction effect is portrayed is especially important to consider with this type of interaction, as the scale alone may underlie the apparent presence or absence of an interaction effect. The second type of interaction, a “nonremovable, pure interaction,” (Figure 1(b)) is characterized by the presence of a genetic effect, which exists only in one of the two environmental exposure groups. In this scenario, the scale is less important to consider than for a removable interaction, although the absolute magnitude of the interaction effect may differ depending on the scale used. The third type of interaction shown here is called a “cross-over” interaction (Figure 1(c)), where a genotype conveys risk of disease in people who are unexposed to the environmental risk factor, but is protective of disease in persons who are exposed to the environmental factor, is probably the rarest, but perhaps most clinically impactful type of interaction. This type of interaction is sometimes called a “nonremovable, cross-over” interaction. In the context of T2D, there are no appropriately replicated examples of cross-over interactions, but in plant genetics, several examples have been documented [102].

## 6. Studies Reporting Gene-Lifestyle Interactions in T2D

Cross-sectional association studies that seek to assess associations or interactions with lifestyle-associated diseases are prone to “labeling” bias, which can differentially affect the accuracy of self-reported lifestyle behaviors in persons with and without a disease diagnosis (or label). For example, because lifestyle is a well-known risk factor for diabetes, people with new onset T2D, when queried about their diet and physical activity levels, may be inclined to overreport consumption of healthy foods and levels of activity, whereas persons who have not been diagnosed with diabetes may report these behaviors more accurately. Thus, cross-sectional studies of lifestyle and T2D are especially prone to bias, particularly when lifestyle exposures are assessed using self-report methods. This caveat applies whether the studies are focused on assessing associations between lifestyle and diabetes or on gene-lifestyle interactions and diabetes. Thus, prospective cohort studies are really required to draw veritable conclusions about the gene-lifestyle interactions and T2D risk, whereas cross-sectional investigations may be sufficient when subclinical diabetes-related traits such as glucose and insulin concentrations are the subject of the interaction analyses. 

Only a handful of published prospective cohort studies have reported interactions between genetic and lifestyle factors in relation to T2D. In a recent study from Sweden, Hindy et al. [103] used a cohort of nearly 25,000 initially nondiabetic Swedish adults to assess interactions between dietary fiber and the *TCF7L2* rs7903146 variant on T2D incidence. In the 12-year follow-up period, 1,649 diabetes events occurred. The authors reported a nominally statistically significant interaction between the *TCF7L2* rs7903146 variant and dietary fiber intake in T2D risk (*P*_interaction_ = 0.049), whereby the odds of developing diabetes conveyed by the *TCF7L2* variant were lower (OR: 1.24, 95% CI 1.04, 1.47) in participants within the highest quartile of dietary fiber intake compared with those in the lowest quartile of fiber intake (OR: 1.56, 95% CI 1.31, 1.86). In the same Swedish cohort, Sondestedt et al, reported statistically significant interactions between the *GIPR* rs10423928 variant and dietary carbohydrate and fat intake in relation to T2D incidence; in these analyses, the odds of diabetes conveyed by the TT genotype were lowest in participants in the highest quintile of dietary carbohydrate intake and in the lowest quintile of dietary fat intake. 

In a separate study from Sweden (the Malmö Preventive Project) involving more than 16,000 initially nondiabetic adults who were followed for 25 yrs (in which ~2,500 diabetes events occurred), Brito et al. assessed gene-physical activity interactions for 17 confirmed T2D loci and T2D. The authors identified one interaction effect that withstood Bonferroni correction at the *HNF1B* locus (rs4430796) [104], in carriers of the low-risk GG genotype, physical activity at baseline was associated with lower rates of diabetes, whereas with each copy of the high-risk A allele, the extent to which physical activity lowered diabetes risk was diminished. The authors also reported interactions between the same variant and physical activity on 2-hour glucose concentrations in 8,860 nondiabetic participants from the same study (*P*_interaction_ = 0.0009). A further two variants (*PPARG* rs1801282 and *CDKN2A/B *rs10811661) showed evidence of interactions with physical activity (*P*_interaction_ = 0.04 and 0.013, resp.) on 2-hour blood glucose concentrations, but there was no statistically robust evidence of interaction on incident T2D. 

Several clinical trials have reported gene-lifestyle interactions on diabetes incidence. The majority of these studies have emerged from the DPP, where both biologic candidate genes [105] and those identified through GWAS have been examined. For example, Florez et al. assessed the interaction of the *TCF7L2* rs7903146 variant and intensive lifestyle modification (versus placebo control) and found no statistical evidence of a gene-treatment interaction (*P*_interaction_ = 0.15) [106]. Mather et al. undertook a comprehensive assessment of common variants within the genes encoding adiponectin (*ADIPOQ*) and its known receptors (*ADIPOR1/2*) none of the *ADIPOQ* variants was directly associated withdiabetesincidence, and although two *ADIPOR1* variants (rs1342387 and rs12733285) were associated with diabetes incidence these variants did not associate with adiponectin concentrations nor did they differ in their effects across treatment arms [107]. Hivert et al. conducted a comprehensive assessment of gene-lifestyle interactions in the DPP by examining 34 previously associated T2D loci. The authors concluded that although in aggregate the SNPs were associated with T2D incidence, there was no difference in effect between lifestyle and control arms, strongly suggesting that these variants do not interact with lifestyle to influence diabetes risk (*P*_interaction_ > 0.05). In two of the initial DPP genetics studies, Moore et al. [108] reported statistically significant gene-lifestyle interactions between the ENPP1 rs1044498 variant and lifestyle intervention on diabetes incidence (*P*_interaction_ = 0.03). Within treatment arm, the Q allele (versus KK homozygotes) was associated with a high hazard ratio (1.38; 95% CI: 1.08–1.76; *P* = 0.009) than in the placebo arm, whereas this effect was apparently abolished by lifestyle intervention.In a separate analysis, Moore et al. [109] reported an interaction between the *CDKN2A/B *rs10811661 variant and lifestyle (versus placebo control) intervention on diabetes incidence, which although this result is only nominally statistically significant (*P*_interaction_ = 0.05), is directionally consistent with the gene-physical activity interaction reported by Brito et al. [104] (see above). 

Thus, while a few studies have provided tentative evidence of gene-lifestyle interactions in T2D, some might argue that few or none have been adequately replicated. It remains implausible that gene-lifestyle interactions do not exist, owing to compelling ecological evidence from ethic comparisons and migration studies, yet in the absence of compelling empirical evidence, it is impossible to draw conclusions about the clinical and public health relevance of specific gene-lifestyle interactions. Future studies that are both larger in scale and which focus on lower frequency gene variants may yield more promising results. It is also possible that the common strategy of focusing primarily on gene variants that have emerged from GWAS of T2D has hindered the discovery of variants that interact with lifestyle; the reasons for why this may be discussed in detail elsewhere [110]. 

## 7. Conclusions

T2D affects almost all developed and developing societies. The major risk factor is abdominal obesity, yet not all those who are obese develop the disease, and not all of those who are at high risk of diabetes remain disease-free by losing weight. The variation in susceptibility to diabetes given different lifestyles is widely believed to be driven by gene variants that interact with environmental factors, yet evidence supporting this view is deficient. Given that it remains highly plausible that gene-environment interactions cause diabetes, and that veritable examples of gene-environment interactions are commonplace in murine and plant models, one should carefully consider why current approaches to studying gene-lifestyle interactions in human diabetes have generally failed to provide concrete support for such relationships. One explanation may be that for common variants, interaction effects are small in magnitude and given the low level of precision with which lifestyle risk factors are generally measured in epidemiology, the sample sizes and mathematical approaches used in studies of interaction afford insufficient statistical power. Indeed, these explanations have fueled a new era of gene-lifestyle interaction studies that are focused on much larger cohort collections and more sophisticated analytical approaches than seen before [111]. It is possible that such studies will yield credible evidence of gene-lifestyle interactions in diabetes and provide clinically-relevant information for the prevention or treatment of this devastating disease.
